# First person – Lozan Sheriff and Reenam Kahn

**DOI:** 10.1242/dmm.048009

**Published:** 2020-12-29

## Abstract

First Person is a series of interviews with the first authors of a selection of papers published in Disease Models & Mechanisms, helping early-career researchers promote themselves alongside their papers. Lozan Sheriff and Reenam Kahn are co-first authors on ‘Alcoholic hepatitis and metabolic disturbance in female mice: a more tractable model than *Nrf2*^−/−^ animals’, published in DMM. Lozan is a postdoctoral research fellow in the lab of Dr Patricia Lalor and Reenam a PhD student in the lab of Prof. Phil Newsome. Both are at the Centre for Liver and Gastrointestinal Research, Institute of Immunology and Immunotherapy, University of Birmingham, Birmingham, UK, investigating the potential of multipotent adult progenitor cells (MAPCs) as a novel therapy for alcoholic steatohepatitis.


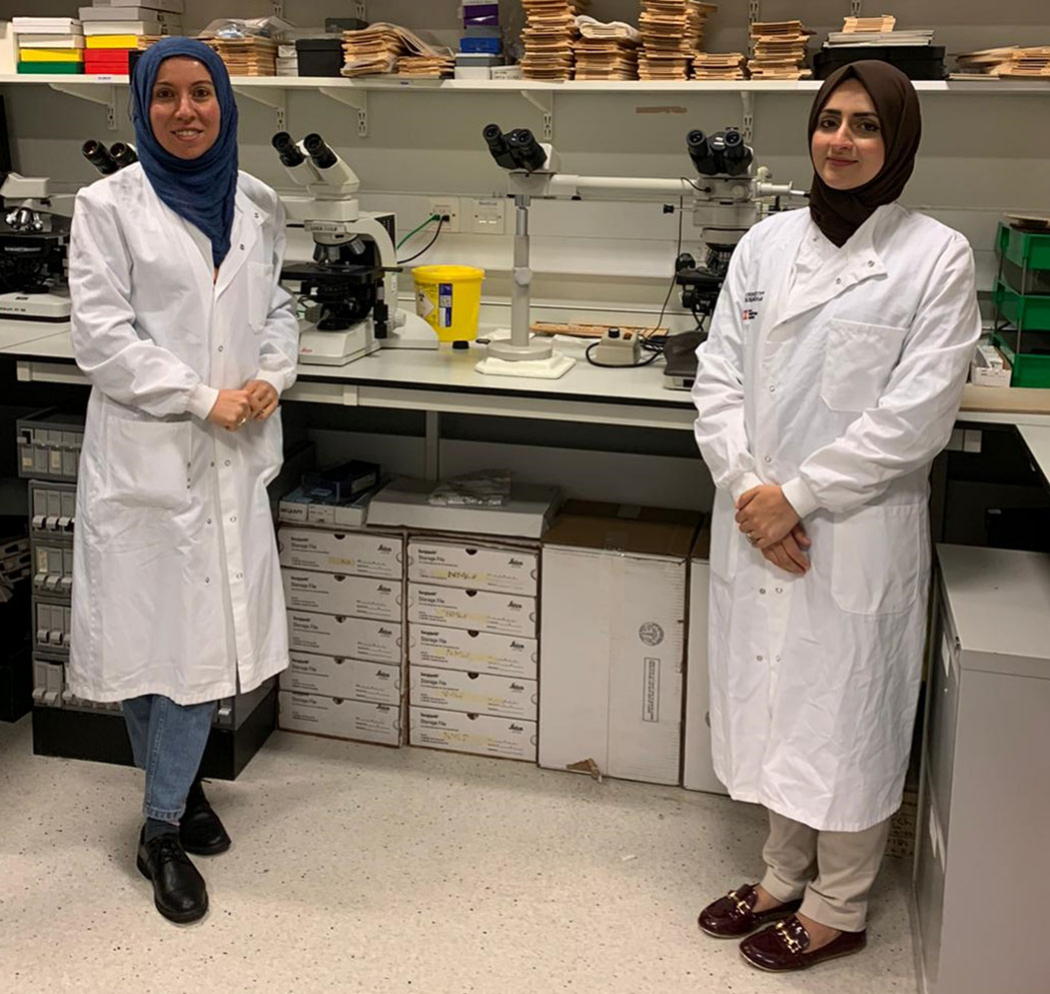


**Lozan Sheriff (left) and Reenam Kahn (right)**

**How would you explain the main findings of your paper to non-scientific family and friends?**

Liver disease accounts for one in ten deaths in people between the ages of 40 and 49, and alcohol-related damage is a major cause. Alcoholic hepatitis (AH) is a severe acute liver injury that is hard to treat, meaning up to 40% of patients currently die. Research into new treatments for AH has been hampered by the lack of animal models that are both practical and accurately recreate human disease. Previous models rely on genetically modified mice that are predisposed to injury. We report a mouse model that reproduces key characteristics of the human disease, including weight loss, signs of inflammation in the liver and the blood, and fatty acid changes in the liver. Importantly, our model uses female mice that – like female humans – can be more susceptible to the damaging effects of alcohol. This means that we do not need to use genetically modified mice and our simple model would have widespread utility for many researchers.

“[…] an animal model of disease that accurately mimics human disease is invaluable for enhancing our understanding of disease pathogenesis […]”

**What are the potential implications of these results for your field of research?**

AH is clearly an area with an unmet clinical need for non-invasive diagnostic techniques and more effective treatments. Having an animal model of disease that accurately mimics human disease is invaluable for enhancing our understanding of disease pathogenesis, and in translating animal studies of novel diagnostic and treatment techniques to clinical settings. Several previous models of AH have been problematic. In models reporting sufficient liver injury, protocols have involved either complex surgical techniques or single-mouse housing, or used end-points, such as mortality, that fall outside local regulatory requirements.

Our model offers a relatively practical method to induce features of AH in mice. This will be helpful for researchers in the field who may wish to use this model.

**What are the main advantages and drawbacks of the model system you have used as it relates to the disease you are investigating?**

Our model recreates several hallmarks of AH. This includes weight loss, hepatic steatosis, hepatic neutrophil infiltration and changes in biochemical markers in serum. In addition, we demonstrated that the alcohol-exposed mice have an altered metabolic profile upon alcohol administration. This will allow us to understand the changes in humans with alcohol-related disease and could permit tailored metabolic support of sick patients in the future.

There are many practical advantages too; our model does not require the use of complex surgery or single mouse housing. Our model uses wild-type animals, which is economically and ethically advantageous compared to the use of genetically modified mice. Further, our protocol does not involve a single high-dose binge of alcohol that typically results in significant physiological signs of illness in exposed animals. Thus, the protocol is more refined from a welfare point of view and has wider applicability across regulatory frameworks.

The main drawback of this model is its acute nature and lack of significant fibrosis. Humans with AH tend to have underlying chronic disease onto which acute injury is superimposed. This still remains a challenge for the scientific community.

**Figure:**
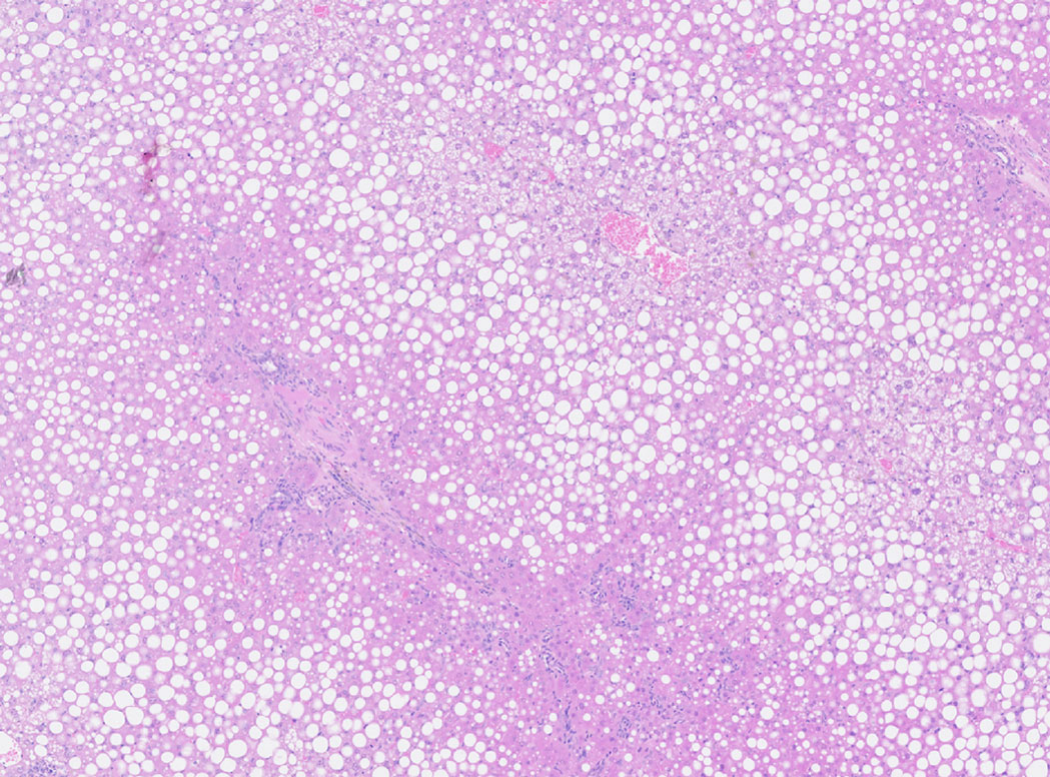
**Haematoxylin and Eosin staining of injured murine liver.**

**What has surprised you the most while conducting your research?**

We were surprised to find that the use of *Nrf2*^−/−^ mice did not confer any advantage in generating a more-severe injury, despite the fact that Nrf2 is a master regulator of antioxidant responses. This may be reflected by the fact we used female animals for our model.

**Describe what you think is the most significant challenge impacting your research at this time and how will this be addressed over the next 10 years?**

Treatment of patients with AH for whom transplantation is not an option is a huge clinical challenge. Improvements in pharmacological therapies will only come from integration of well-validated human cohort data and use of representative animal models. In our opinion, the most significant challenge for research into alcoholic liver disease is to produce reproducible murine models of liver disease, in which there is hepatic fibrosis and replicative senescence to mirror the human picture. Importantly, these models need to recreate the typical acute or chronic nature of injury – but in a manner that does not compromise animal welfare and is acceptable to regulatory ethical bodies internationally.

**What changes do you think could improve the professional lives of early-career scientists?**

We think that more national and international collaboration to share research samples and databases, particularly in the ‘omics’ era, would help researchers to make more efficient use of a smaller number of animals in research, as well as saving researchers’ time and costs. In particular, partnerships between clinical researchers and scientists will improve the translational potential of new discoveries.

**What's next for you?**

We now plan to refine the existing model by incorporating replicative senescence to allow us to test some of the novel therapeutic molecules that have arisen from our studies in the Centre for Liver and Gastrointestinal Research at the University of Birmingham. Our ultimate goal is to produce data that can be used to help human studies to address the clinical needs of patients with AH.
